# The effects of dietary edible bird nest supplementation on learning and memory functions of multigenerational mice

**DOI:** 10.1002/brb3.1817

**Published:** 2020-09-04

**Authors:** Obaidullah Mahaq, Mohd Adha P. Rameli, Marilyn Jaoi Edward, Nursyuhaida Mohd Hanafi, Saleha Abdul Aziz, Hasliza Abu Hassim, Mohd Hezmee Mohd Noor, Hafandi Ahmad

**Affiliations:** ^1^ Department of Veterinary Preclinical Sciences Faculty of Veterinary Medicine Universiti Putra Malaysia UPM Serdang Selangor Darul Ehsan Malaysia; ^2^ Department of Veterinary Preclinical Science Faculty of Veterinary Medicine Shaikh Zayed University Khost Afghanistan; ^3^ Agro‐Biotechnology Institute (ABI) National Institutes of Biotechnology Malaysia (NIBM), c/o MARDI Headquarters Serdang Malaysia; ^4^ Department of Veterinary Pathology and Microbiology Faculty of Veterinary Medicine Universiti Putra Malaysia UPM Serdang Selangor Darul Ehsan Malaysia; ^5^ Laboratory of Sustainable Animal Production and Biodiversity Institute of Tropical Agriculture and Food Security University Putra Malaysia UPM Serdang Selangor Darul Ehsan Malaysia; ^6^ University Agriculture Park Universiti Putra Malaysia UPM Serdang Selangor Darul Ehsan Malaysia

**Keywords:** brain gene expression, edible bird nest, learning and memory, multigenerational mice, sialic acid, synaptic vesicle

## Abstract

**Introduction:**

Edible bird nest (EBN) is a natural food product produced from edible nest swiftlet's saliva which consists of glycoproteins as one of its main components; these glycoproteins contain an abundant of sialic acid. The dietary EBN supplementation has been reported to enhance brain functions in mammals and that the bioactivities and nutritional value of EBN are important during periods of rapid brain growth particularly for preterm infant. However, the effects of EBN in maternal on multigeneration learning and memory function still remain unclear. Thus, the present study aimed to determine the effects of maternal EBN supplementation on learning and memory function of their first (F1)‐ and second (F2)‐generation mice.

**Methods:**

CJ57BL/6 breeder F0 mice were fed with EBN (10 mg/kg) from different sources. After 6 weeks of diet supplementations, the F0 animals were bred to produce F1 and F2 animals. At 6 weeks of age, the F1 and F2 animals were tested for spatial recognition memory using a Y‐maze test. The sialic acid content from EBN and brain gene expression were analyzed using HPLC and PCR, respectively.

**Results:**

All EBN samples contained glycoprotein with high level of sialic acid. Dietary EBN supplementation also showed an upregulation of GNE, ST8SiaIV, SLC17A5, and BDNF mRNA associated with an improvement in Y‐maze cognitive performance in both generations of animal. Qualitatively, the densities of synaptic vesicles in the presynaptic terminal were higher in the F1 and F2 animals which might derive from maternal EBN supplementation.

**Conclusion:**

This study provided a solid foundation toward the growing research on nutritional intervention from dietary EBN supplementation on cognitive and neurological development in the generation of mammals.

## Graphical abstract

The effects of EBN on learning and memory function of multigenerational mice.

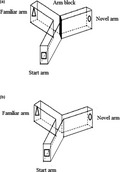

## 1 INTRODUCTION

Edible bird nest (EBN) produced from the saliva of edible nest swiftlets (*Aerodramus fuciphagus)* which are commonly found in the South‐East Asia countries including Malaysia. EBN has been reported worldwide as an effective health supplementation and food product as well as beauty enhancer (Hao & Rahman, 2016; Wong, 2013). The major nutrient components in the nest are glycoproteins, carbohydrates, and essential trace elements such as calcium, sodium, magnesium, zinc, and iron (Norhayati, Azman, & Wan Nazaimoon, 2010). However, it is established that the glycoprotein from EBN is found to contain sialic acid (Aswir & Nazaimoon, 2011; Norhayati et al., 2010; Xie et al., 2018). In fact, it has been reported that sialic acid from EBN can facilitate the development of brain and intelligence (Wang, 2009, 2012; Xie et al., 2018). Although sialic acid levels have been associated with the EBN, a more comprehensive study should be carried out to investigate the role of sialic acid from EBN especially in the improvement of learning and memory functions on the preterm infant.

The newborn infant's growth and development requires outstanding demands on the nutrients supply. Any deficit of nutrient has profound effects on somatic growth, organ structural and functional development, especially in the brain. One of the important nutrients is sialic acid (also known as N‐acetylneuraminic acid), a nine‐carbon sugar that is a structural and functional component of brain gangliosides (Wang, McVeagh, Petocz, & Brand‐Miller, 2003). The gangliosides induced by sialic acid may play a role in the structural and functional establishment of neurotransmitter in the synaptic vesicle (Reigada et al., 2003) and altering existing synaptic morphology (Morgan, Kuyatt, & Fink, 1985; Morgan, Oppenheimer, & Winick, 1981; Morgan & Winick, 1981). The mechanism as to how sialic acid mediates the brain function system is yet to be discovered; it is envisioned that the gene expression analysis may give additional information with regard to the possible involvement of sialic acid.

It has been reported that genes related to learning and memory functions were found overexpressed in animal's brain treated with sialic acid supplementation (Kurosawa, Yoshida, Kojima, & Tsuji, 1997; Wang et al., 2007). Several genes such as UDPN‐acetylglucosamine‐2‐epimerase/N‐acetylmannosamine kinase (GNE) and α‐2,8‐sialyltransferase (ST8SiaIV) mRNA involved in signal transduction and synaptic plasticity were overexpressed in piglet's brain receiving sialic acid‐enriched diet (Wang et al., 2007). In such studies, ST8SiaIV mRNA expression was increased in both neonatal and adult's brain when they were supplemented with sialic acid (Kurosawa et al., 1997; Yoshida, Kojima, & Tsuji, 1995). The relative mRNA expression levels of Solute Carrier Family 17 Member 5 (SLC17A5) gene were upregulated in the hippocampus of pigs after supplementation with milk rich in sialyllactose (Obelitz‐Ryom et al., 2019). Moreover, maternal EBN supplementation during pregnancy and lactation periods enhanced the levels of sialic acid in maternal mouse milk, which was associated with an increased level of brain‐derived neurotrophic factor (BDNF) gene among their generations (Xie et al., 2018).

An expression of some genes resulted from dietary sialic acid supplementation has a marked influence on the brain physiological processes such as cell adhesion and signal transduction toward to proper brain cognitive development. However, the upregulation of brain gene expression by EBN supplementation remains controversial since the content of sialic acid varied in the EBN sources from different localities. In addition, brain cognitive functions have been linked to different genetic alterations which can be transferred across generations early in life (Alese & Mabandla, 2019). The transfer process, also referred to as epigenetics changes, is involved with a chromatin network modifications and alteration of DNA methylation (Fagiolini, Jensen, & Champagne, 2009). Thus, the alteration of DNA methylation may interact with other epigenetic changes to regulate the expression of brain genes that are involved in a wide range of learning and memory functions.

The long generation gap in humans, as well as the relative difficulty in quantifying cognitive function development in human neonates, remains a major obstacle in studying the effects of dietary EBN supplementation associated with neural function development. The content of sialic acid from EBN is critical under conditions of rapid growth of the infant brain, particularly during the neuron development in several generation mammals. The effects of sialic acid on brain cognitive function are well known; however, little is known about the cellular changes and the mechanism effects of sialic acid from EBN‐supplemented diet associated with cognitive function in multigeneration mammals. Previously, we confirmed that cognitive deficits after multigenerational maintenance on omega‐3 fatty acid‐deficient diet can be prevented by 16 weeks of dietary repletion with omega‐3 fatty acids (Hafandi et al., 2014). Thus, the findings arising from this study may affect the way in which dietary modifications such as addition of EBN supplementation so as to improve brain cognitive function in the next‐generation mammals.

## 2 MATERIALS AND METHODS

### 2.1 Edible bird nest (EBN) collection and preparation

A total of three raw unprocessed EBN samples from three different states of Malaysia (e.g., North and South Peninsular Malaysia, and Borneo Sabah) were collected during the breeding season of edible nest swiftlet from April to August 2017. The samples were manually cleaned from gross dirt, and the visible feathers were removed using forceps. The commercial EBN was purchased from a retail outlet (Dingshen Imperial Birdnest). The EBN was finely grounded using an electrical grinder. Briefly, 10 mg/kg of dried EBN was immersed in the distilled water for 3 hr. Then, the soaked EBN in the water was boiled for 30 min with the solid–liquid ratio at 1:10 (g/ml). This solution was used to feed the individual animals. In addition, the sialic acid content from EBN was determined at SIRIM Berhad, Malaysia, using the high‐performance liquid chromatography (HPLC).

### 2.2 Animals

**Figure 1:**
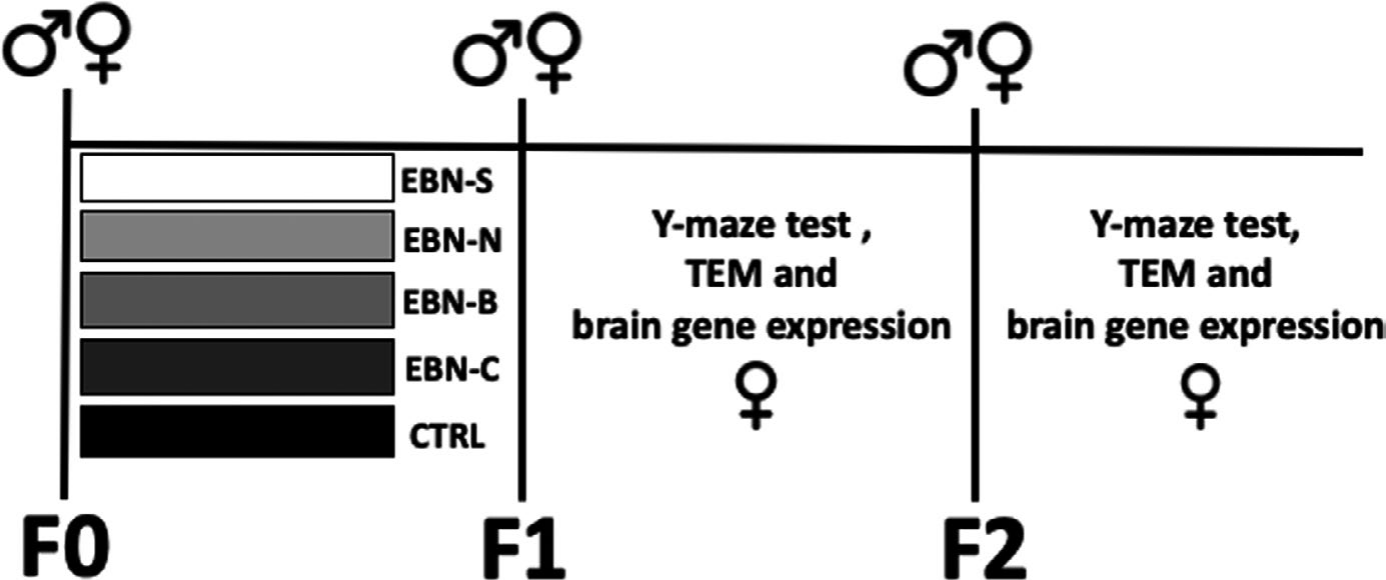
Experimental design of the dietary EBN supplementation on the maternal mice (F0), and their effects on the cognitive function, TEM, and brain gene expression of F1 and F2 generations. F0, F0 generation; F1, first generation; F2, second generation; TEM, transmission electron microscopy; EBN, edible bird nest; EBN‐S, EBN from South; EBN‐N, EBN from North; EBN‐B, EBN from Borneo; EBN‐C, EBN commercial; CTRL, Control

CJ57B/6 breeder's mice at 4 weeks of age (*n* = 60, 20 males and 40 females) were purchased from the Biosystem Corporation, Singapore. These breeders were considered as F0 generation and were used to produce the first (F1)‐ and second (F2)‐generation of mice (Figure 1). One week before mating, the breeder mice were divided into four groups and were treated with EBN from different sources by oral gavage (10 mg/kg); EBN was sourced from South Peninsular Malaysia (EBN‐S; *n* = 8), North Peninsular Malaysia (EBN‐N; *n* = 8), Sabah (Borneo) Malaysia (EBN‐B; *n* = 8), and EBN Commercial (EBN‐C; *n* = 8). The mice given tab water were categorized as control group (CTRL; *n* = 8). All animals were given food pellets and water ad libitum. After six weeks of treatment diet, all animals were mated in the Animal House, Faculty of Veterinary Medicine, Universiti Putra Malaysia, and the treatment diets were given continuously until the female mice were pregnant. It was estimated that the female mice gave birth to approximately eight female pups of F1 generation in each treatment group and a control group (*n* = 40). After weaning at week 3, the breeder mice were removed from the cage and the F1 female mice were given free access pellets and water ad libitum. At 6 weeks of age, the F1 female mice were tested on spatial recognition memory using a Y‐maze test. Our previous finding reported that 3 weeks of dietary with omega‐3 fatty acid supplementation improved cognitive function in mice (Nur Farhana et al., 2015). Therefore, in this current study, at 6 weeks of age is sufficient time to evaluate the mice on cognitive performance. Then, at 8 weeks of age, the F1 female mice were mated to produce the F2 generation of mice. At 6 weeks of age, the F2 female mice were tested on spatial recognition memory similar to F1 female mice. At the end of the test, the F1 and F2 female mice were euthanized and their brains were processed for histological and gene expression study.

All animal testing was approved by the Institutional for Animal Care and Use Committee (IACUC), Universiti Putra Malaysia (UPM/IACUC/AUP‐R092/2017).

### 2.3 Learning and memory functions by the Y‐maze test

**Figure 2:**
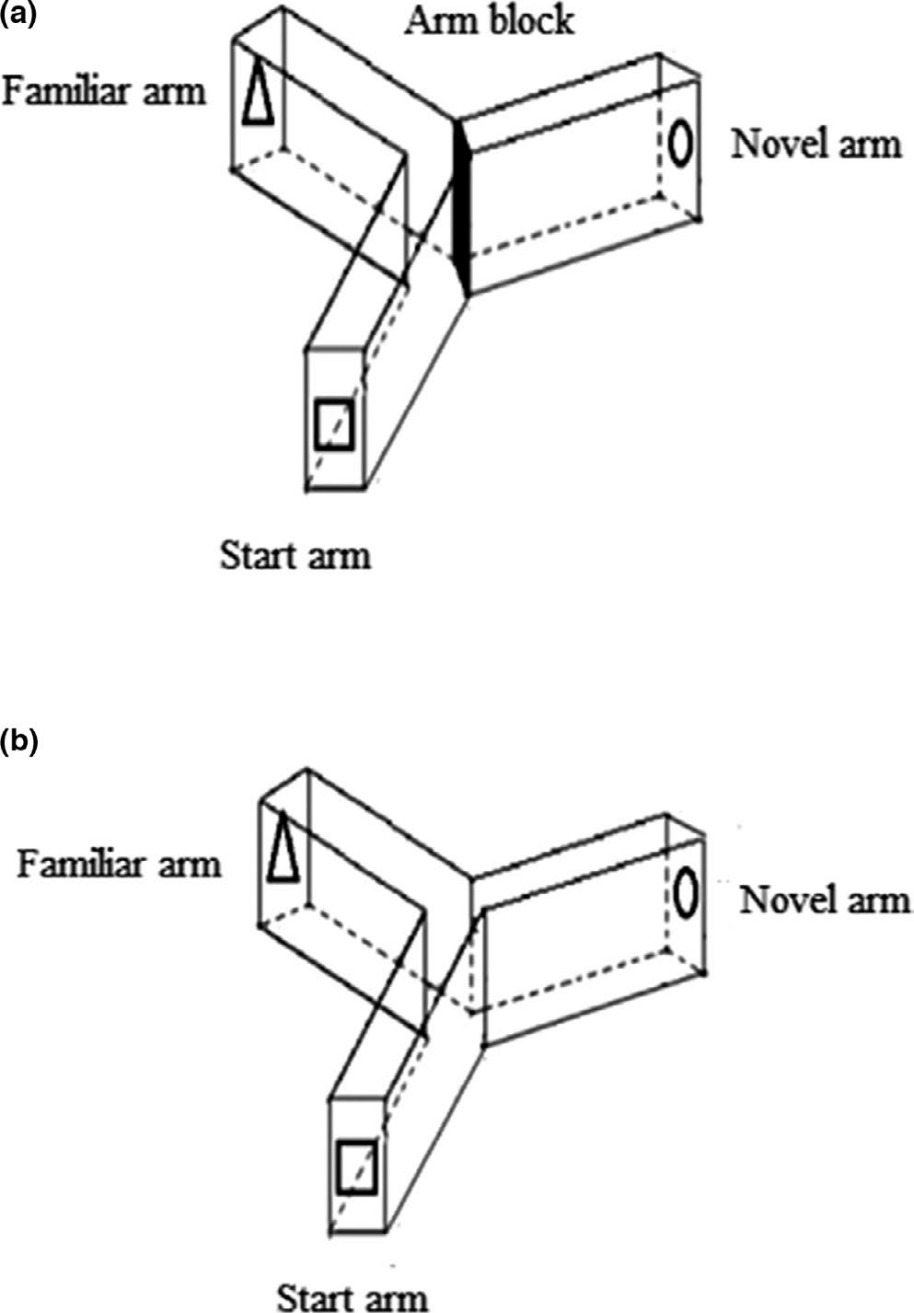
Schematic representation of the Y‐maze test with (a) the novel arm blocked with a sliding partition during the first trial, but open during the second trial (b). Different visual cues (square, circle, and triangle) were placed on the external maze walls

The learning and memory functions of mice were tested using the Y‐maze which was described previously (Hafandi et al., 2014). The Y‐maze had three identical arms of equal size: the start arm, in which the mouse is first placed (always open); the familiar arm (always open); and the novel arm, which was blocked during the first trial but opened during the second trail (Figure 2). Different visual cues were placed on the wall at the end of each arm of the maze. The Y‐maze testing consisted of two trials separated by an interval of 1 hr. The first trial was 10 min in duration and allowed the mouse to explore only two arms (the start and familiar arms) of the maze, with the third arm (novel arm) blocked. After 1 hr, the second trial was conducted; mice were placed in the same starting arm as in trial 1, with free access to all three arms for 5 min. Trials were recorded by using a ceiling‐mounted camera. Recordings were then watched to count the number of entries and the time spent in each arm. Y‐maze performance was favorable when the number of entries and time spent in the novel arm were greater than those in the other arms. The total number of arm entries and time (in seconds) spent in the novel arm are indicator of spatial working memory (Hafandi et al., 2014; Nur Farhana et al., 2015).

### 2.4 Transmission Electron Microscopy (TEM) brain neuron histological changes

The brain hippocampus was prepared of about 1 mm^3^ in diameter and fix with 2.5% glutaraldehyde for 24 hr. Then, the samples were washed three times with buffer solutions for 10 min each and postfix for 2 hr with 1% osmium tetraoxide. The samples were rinsed and dehydrated in ascending series of acetone wash (35%, 50%, 75%, and 95%) for 10 min at each concentration. The last part of dehydration was done by soaking three times in 100% acetone for 10 min each time. The dehydrated samples were infiltrated with resin‐acetone mixture. After infiltration, the samples were embedded in the beam capsule or flat embedder filled with the resin mixture and polymerize in oven at 60°C for 24–48 hr. Semithin sections (1 μm thick) were cut by using ultramicrotome (Ultracut E, Reichert‐Jung, Austria). Then, the semithin sections were stained with Toluidine blue and viewed under light microscope in order to select the region of interest prior to the ultrathin sectioning. Ultrathin sections of 80–90 nm were cut and mounted onto 200‐mesh copper grids. The sections were stained with uranyl acetate and lead citrate for 10 min and washed with double distilled water. The stained sections were examined using transmission electron microscope (Hitachi H7100, Japan) at 80 kV for ultrastructural studies.

### 2.5 RNA extraction and real‐time RT‐PCR

Tissue samples of hippocampus (~20 mg) were collected and extracted by using RNeasy Mini Kit Qiagen, German. The quality and quantity of extracted RNA were analyzed using Thermo Scientific NanoDrop2000c. The final concentration of cDNA 50 ng/µl was produced from the total RNA from each sample of using One Taq^®^ RT‐PCR kit (New England Biolabs) according to manufacturer instructions. The qRT‐PCR assays for relative quantification of the respective gene expression (e.g., GNE, SLC17A5, ST8SiaIV, and BDNF) in each sample were performed using an iTaq Universal SYBR Green Supermix from Bio‐Rad. The PCR primers set for the all genes were adapted from the previous study (Duncan, Raymond, Fuerholz, & Sprenger, 2009; Wang et al., 2007; Xie et al., 2018) and were synthesized by first base according to the cDNA sequences obtained from the Gene Bank TM database for mouse. The relative expression ratios were normalized to β‐actin and GADPH, and the sequences of primers are presented in Table 1.

**Table 1:** The primer and the accession number of genes

Genes	Primers (F; Forward and R; Reverse)	Amplicon Size (bp)
Housekeeping Gene		
β‐actin	F = 5‐TTGTGATGGACTCCGGAGAC‐3 R = 5‐TGATGTCACGCACGATTTCC‐3	173
GAPDH	F = 5‐CCACCCAGAAGACTGTGGAT‐3 R = 5‐CACATTGGGGGTAGGAACAC‐3	186
Genes of Interest		
GNE	F = 5‐GCATGAAGGGTGAAATCGTT‐3 R = 5‐ATGCAGCACAACTCCTTCCT‐3	188
SLC17A5	F = 5‐GGCTACATCGTCACCCAGAT‐3 R = 5‐AACACCCTCTCCCAGTCCTT‐3	180
ST8SiaIV	F = 5‐TGTGCAAAGAGCATTTGGAG‐3 R = 5‐TTCGCACTTGCAGTTTGTTC‐3	176
BDNF	F = 5‐GGTATCCAAAGGCCAACTGA‐3 R = 5‐CTTATGAATCGCCAGCCAAT‐3	183

During initial optimization, the exact primer concentrations and PCR conditions were determined. Following optimization experiments, the assays of total volume of 20 µl reaction mix consisting of an equal concentration of RNA, 10 µl of iTaq Universal SYBR Green Supermix from Bio‐Rad with 10 nM of each forward and reverse primer were performed. The standard curve and melting curve were run in CFX96 real‐time PCR machine (Bio‐Rad) with amplification involving one cycle at 95°C for 5 s and primer annealing at 55.8°C for 30 s. Melt curve analysis was carried out after amplification within temperature range of 65°C to 95°C with 0.5°C increments at 5 s/step.

Two‐step qRT‐PCR analyses were used to determine the expression rate for gene of interest. All samples were amplified on the same plate for every primer pair to ensure equal amplification conditions, and no‐template controls with water instead of RNA templates were also included as negative controls. Each sample was then run in triplicate, and their results were documented as cycle threshold (C_T_) values. Expression rate was determined using the relative quantification method (2‐ΔΔCt). The difference between the threshold value (Ct) of gene of interest and housekeeping gene was calculated. Difference in Ct values (ΔCt) was obtained for each sample was averaged and subtracted from the mean value of ΔCt of control sample. Then, the value of ΔCt difference (ΔΔCt) obtained was used to determine the relative expression rate of each gene in each sample.

### 2.6 Statistical analysis

All data were expressed as mean ± *SEM* (standard error) and analyzed with the statistics software package (SPSS) for Windows version 25. The assumption of normality and homogeneity of variances were assessed by Shapiro–Wilk's test and Levene's test, respectively. One‐way ANOVA was used to compare the effects of different maternal EBN supplementation on the cognitive performance of F1 and F2 animals. The significance of the difference between comparisons was determined by using Tukey's HSD test. All statistical tests were performed at 95% confident level, and *p* < .05 was considered to be significant.

## 3 RESULTS

### 3.1 Sialic acid content of EBN

The retention time and HPLC analysis of sialic acid content from different EBN sample as shown in Table 2. The N‐acetylneuraminic acid was detected using a standard compound after 20 min of HPLC analysis. The content of N‐acetylneuraminic acid in 100 g of EBN from North and South Peninsular Malaysia was 3.15 ± 0.34% and 2.97 ± 1.63%, respectively, which were higher compared to EBN from Sabah (2.02 ± 1.76%) and commercial EBN (1.17 ± 0.10%).

**Table 2:** The retention time and HPLC results of each edible bird's nest

Edible bird nest (EBN)	Relative retention time (min)		HPLC parameters (Weight of EBN = 0.01015 g)		
	Chromatogram of sialic acid (N‐Acetylneuraminic acid) standard	Chromatogram of EBN	Linearity	Correlation coefficient (*R* ^2^)	Content of sialic acid (%)
EBN‐S	6.40	6.33	*Y* = 2,629.2*x* – 7,030.6	.9998	2.97 ± 1.63
EBN‐N	6.40	6.33	*Y* = 2,629.2*x* – 7,030.6	.9998	3.15 ± 0.34
EBN‐B	6.44	6.42	*Y* = 2,599.6*x* + 6,756.5	.9998	2.02 ± 1.76
EBN‐C	6.40	6.33	*Y* = 2,629.2*x* – 7,030.6	.9998	1.17 ± 0.10

### 3.2 Y‐maze performance

**Figure 3:**
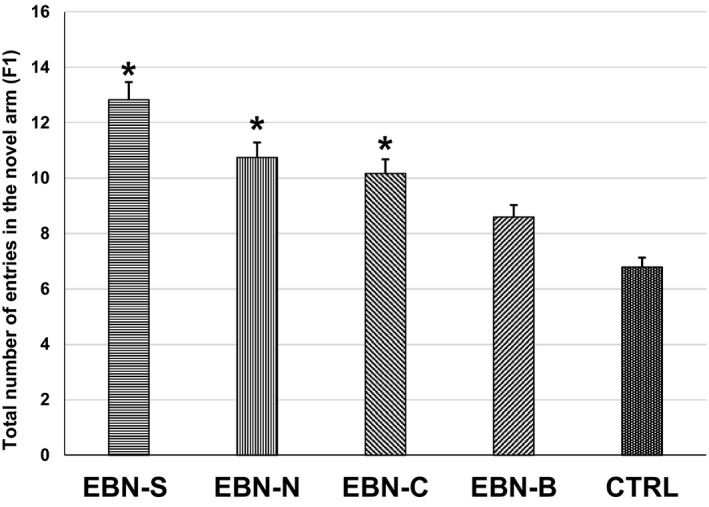
The effects of dietary EBN supplementation of F1 animals on the total number of arm entries in the novel arm. Values are expressed as mean ± *SEM*. Significant difference indicated with superscript (*) and *p* < .05 were considered to be significant

**Figure 4:**
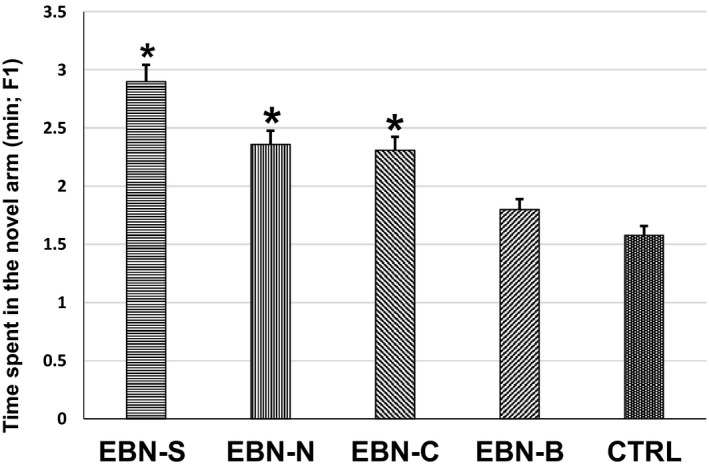
The effects of dietary EBN supplementation of F1 animals on the time spent in the novel arm (min). Values are expressed as mean ± *SEM*. Significant difference indicated with superscript (*) and *p* < .05 were considered to be significant

One‐way ANOVA showed a significant interaction (*F*_4,16_ = 8.251, *p* < .05) between treatment groups of F1 animals on the number of entries in the novel arm (Figure 3). Post hoc comparisons using Tukey HSD test indicated that the F1 animals from maternal EBN‐S supplementation had significantly higher number of entries in the novel arm (12.83 ± 0.22) compared to EBN‐B (8.60 ± 1.11) and CTRL (6.79 ± 0.95). However, the number of entries in the novel arm was not significantly different among the EBN‐S, EBN‐N, and EBN‐C groups. Meanwhile, the time spent in the novel arm of F1 animals was significantly different among groups (*F*_4,16_ = 7.100, *p* < .05; Figure 4). The time spent in the novel arm of F1 animals from maternal EBN‐S supplementation was significantly higher (2.90 ± 0.02 min) compared to EBN‐B (1.92 ± 0.22 min) and CTRL (1.58 ± 0.25 min). However, the time spent in the novel arm was not significantly different among the EBN‐S, EBN‐N, and EBN‐C groups.

**Figure 5:**
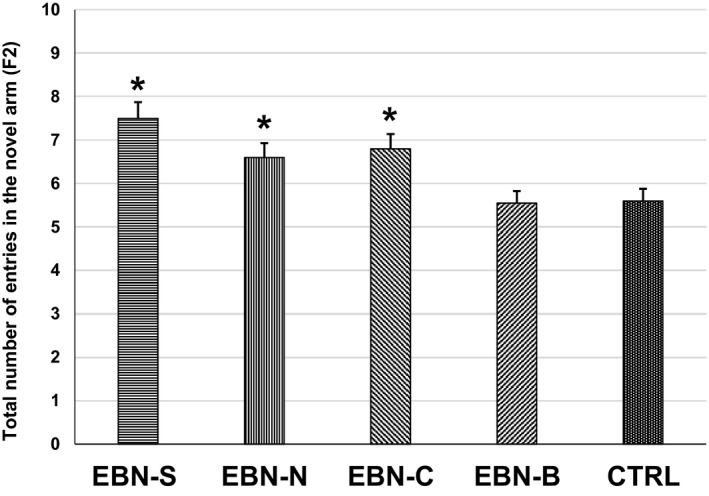
The effects of dietary EBN supplementation of F2 animals on the total number of arm entries in the novel arm. Values are expressed as mean ± *SEM*. Significant difference indicated with superscript (*) and *p* < .05 were considered to be significant

**Figure 6:**
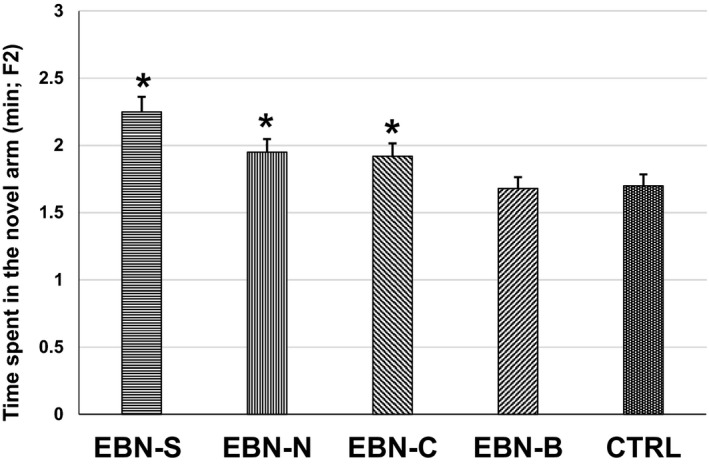
The effects of dietary EBN supplementation of F2 animals on the time spent in the novel arm (min). Values are expressed as mean ± *SEM*. Significant difference indicated with superscript (*) and *p* < .05 were considered to be significant

One‐way ANOVA showed a significant interaction (*F*_4,27_ = 5.361, *p* < .05) among treatment groups of F2 animals on the number of entries in the novel arm (Figure 5). The F2 animals from maternal EBN‐S supplementation had significantly higher number of entries (7.43 ± 0.37) in the novel arm than EBN‐B (5.52 ± 0.26) and CTRL (5.61 ± 0.33). However, there were no significant differences on the number of entries in the novel arm among EBN‐S, EBN‐N, and EBN‐C groups. Similarly, the time spent in the novel arm of F2 was significantly different among groups (*F*_4,27_ = 2.845, *p* < .05; Figure 6). The time spent in the novel arm was significantly higher in the F2 animals from maternal EBN‐S supplementation (2.05 ± 0.07 min) compared to EBN‐B (1.68 ± 0.12 min) and CTRL (1.72 ± 0.12 min). However, the time spent in the novel arm of the F2 animals from maternal EBN‐N and EBN‐C supplementation did not differ significantly from the EBN‐S.

### 3.3 TEM brain neuron histological changes

**Figure 7:**
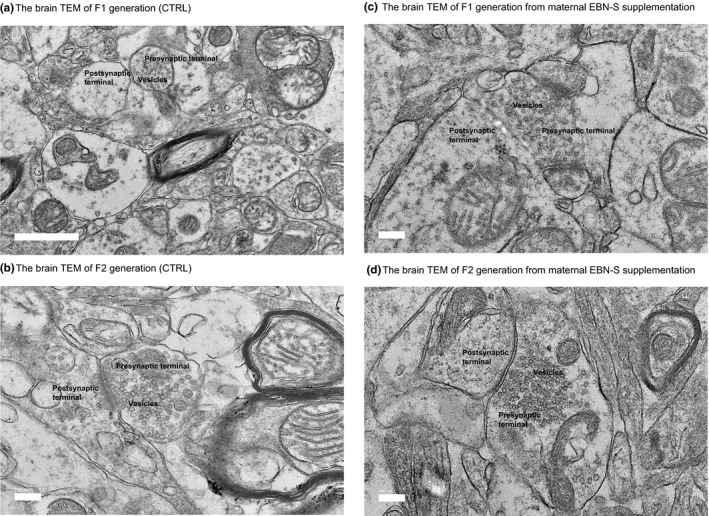
(a, b, c, and d) Transmission electron microscopy (TEM) shows the densities of vesicle, presynaptic, and postsynaptic terminal. The scale bar represents 0.5 mm. (a) The brain TEM of F1 generation (CTRL). (b) The brain TEM of F2 generation (CTRL). (c) The brain TEM of F1 generation from maternal EBN‐S supplementation. (d) The brain TEM of F2 generation from maternal EBN‐S supplementation

Figure 7a–d shows the synaptic vesicle distribution on the pre‐ and postsynaptic areas of CA1 hippocampus region. Qualitatively, the density of synaptic vesicles of both F1‐ and F2‐generation mice from maternal EBN‐S supplementation was more dense as compared to F1 and F2 control mice. However, the density of synaptic vesicle on EBN‐N, EBN‐C, and EBN‐B both in F1 and F2 mice is parallel to EBN‐S (data were not shown).

### 3.4 Brain gene mRNA expression

**Figure 8:**
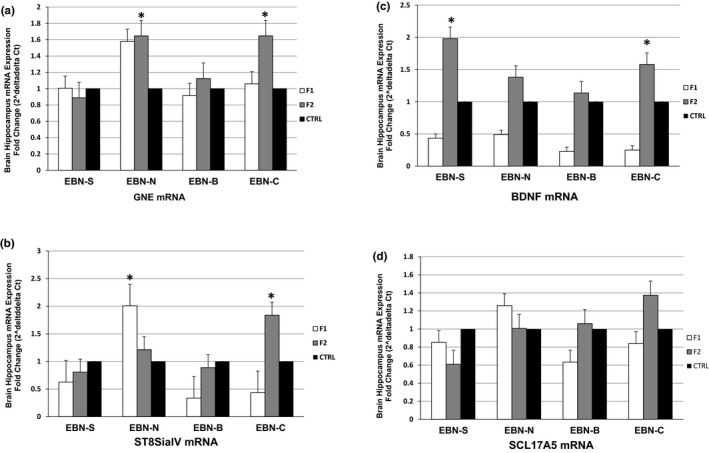
(a, b, c, and d) Expression of GNE, ST8SiaIV, BDNF, and SCL17A5 mRNA in the brain hippocampus of treatment groups compared to the CTRL group. Results are normalized to the expression of GAPDH and β‐actin. Values indicated by * show significant differences compared with the CTRL group at *p* < .05. F1, first generation; F2, second generation

Figure 8 shows the upregulation level of GNE, ST8SiaIV, BDNF, and SLC17A5 mRNA genes in the hippocampus area of F1 and F2 animals compared to the control group. The GNE mRNA level was significantly higher in the F2 animals which from maternal EBN‐N (1.64‐fold change; *p* < .05) and EBN‐C (1.65‐fold change; *p* < .05) supplementation, compared to the control group. The ST8SiaIV mRNA was significantly higher in the F1 group with EBN‐N (2.00‐fold change; *p* < .05) and the F2 group with EBN‐C (1.84‐fold change; *p* < .05) supplementation. In addition, the BDNF mRNA was significantly higher in the F2 group with EBN‐S (1.98‐fold change; *p* < .05) and EBN‐C (1.60‐fold change; *p* < .05) supplementation. However, there were several variation regulations on the SLC17A5 mRNA both in the F1 and F2 groups.

## 4 DISCUSSION

The current study showed the content of sialic acid in 100 g of EBN samples ranged from 1.17% to 3.15%. However, Norhayati et al. (2010) reported sialic acid content in EBN obtained from North, South, and East Peninsular Malaysia was ranged from 0.70% to 1.50%, while Marni et al. (2014) reported EBN samples obtained from Johor (South Peninsular Malaysia) and Kelantan (East Peninsular Malaysia) had high sialic acid, ranging from 9.5% to 13.5%. The sialic acid contents in EBN obtained from different locations in Malaysia were found to vary; this could be due to a number of factors such as different breeding sites, types of habitat, environmental surroundings, and diet or food available for *Aerodramus* (Hamzah, Hulwani Ibrahim, Hussin, Hashim, & Lee, 2013; Huda, Shukri, Nawi, Abdullah, & Man, 2018; Khalid, Rashed, Aziz, & Ahmad, 2019; Norhayati et al., 2010).

The content of sialic acid in all EBN groups in this study was found to improve learning and memory functions in the F1 and F2 animals. This was shown by the higher number of entries and time spent in the novel arm of the Y‐maze task performance compared to the control animals. This was probably due to the maternal mice were given EBN which contained high sialic acid that might had positive effect on the cognitive performance of their F1 and F2 offspring. These results indicated that the sialic acid from EBN was synthesized through the placenta and maternal milk which contributed to the neuronal transmission and synaptic plasticity development in their offspring (Oliveros et al., 2018; Wang et al., 2003; Xie et al., 2018). In addition, the synthesis of sialic acid in the F1 and F2 offspring improved the concentration of ganglioside bound and protein bound in the brain, suggesting that an increased sialylation on gangliosides mediates the learning and memory responses (Karim & Wang, 2006). In fact, the metabolism of sialic acid from EBN produced several neuraminic acids (e.g., N‐acetyl‐D‐galactosamine, N‐acetyl‐D‐glucosamine, and N‐acetylneuraminate) that are integral structural and functional components of the nervous system (Karim & Wang, 2006; Khalid et al., 2019; Yu‐Qin et al., 2000). Therefore, the ability of F1 and F2 mice to complete the Y‐maze learning task suggested that the intake of exogenetic sialic acid from EBN is an essential micronutrient for optimal brain metabolism and cognitive development in multigeneration mice.

Dietary EBN supplementation for maternal mice showed an increase in the densities of synaptic vesicle in the pre‐ and postsynaptic CA1 hippocampus region on their F1 and F2 generations. This finding is consistent with previous studies that showed the numbers of neurons on CA1, CA3, and DG regions of the hippocampus were significantly higher in mice treated with EBN rich in sialic acid (Xie et al., 2018). The higher densities of synaptic vesicles as shown in our study were in parallel with an improvement in the Y‐maze task performance especially in the F1 and F2 animals. A possible explanation is that the sialic acid in EBN plays an important role at the synapses which can modulate the neurotransmission from the vesicle, via interactions calcium ion channel and chemical messages (Palmano, Rowan, Guillermo, Guan, & McJarrow, 2015; Rahmann, 1995; Wang, 2012). In fact, the synthesis of sialic acid in the neurons leads to altered synaptic plasticity required for memory consolidation (Foley et al., 2003; Wartman & Holahan, 2014). Thus, this probably indicated that EBN supplementation promotes the development of hippocampal neurons, which in turn affects the number and quality of synaptic vesicles connections especially during learning and memory processes.

There are some variations in the regulations of the GNE, ST8SiaIV, BDNF, and SLC17A5 gene mRNAs in the F1 and F2 generation which indicated that dietary EBN supplementation from maternal influenced the regulation of brain genes associated with the sialic acid metabolism. The regulation of brain genes expression by dietary EBN supplementation is due to the synthesis of the polysialic acid from EBN which play critical roles in modifying the interactions of the neural cell adhesion molecules (NCAM) (Schnaar, Gerardy‐Schahn, & Hildebrandt, 2014). This could suggest that GNE, ST8SiaIV, BDNF, and SLC17A5 gene mRNA in the F1 and F2 mice function simultaneously to increase the synthesis of polysialic acid on NCAM during the period of high sialic acid demand such as during brain growth and development. Previous findings had also reported that the regulation of brain gene expression by dietary omega‐3 fatty acids is due to the interactions of fatty acids with the ligands that bind to response factors acting on cis‐regulatory elements of the gene, which finally turn on or off mRNA synthesis (Kitajka et al., 2004; Nur Farhana et al., 2015).

The current study showed EBN supplementation in maternal mice increased the BDNF gene in both generations due to the ability of EBN compounds to activate the extracellular signal‐regulated kinase and signaling pathway. This pathway may lead to the activation of the cyclic adenosine monophosphate response element binding protein, which is a transcription factor that can increase the expression of a neurotrophins such as BDNF (Meeusen, 2014). Indeed, BDNF promotes neurons survival, maturation, and synaptic functions and involved in several aspects of learning and memory functions (Bathina & Das, 2015; Buchman et al., 2016; Fernandes et al., 2011). It is suggested that the BDNF can induce neural plasticity and mitochondrial biogenesis, and hence has the ability to improve the hippocampal neurogenesis during pre‐ and postnatal development. Hence, the improvement in learning and memory functions in the Y‐maze task in F1 and F2 mice as shown in this study was due to the synthesis of BDNF gene resulted from the dietary EBN supplementation in maternal mice especially during pregnancy and lactation periods.

Our finding also suggested that an upregulation of BDNF gene in the hippocampus was due to the changes in DNA methylation during learning and memory formation of Y‐maze task in the F1 and F2 generations. In fact, DNA methylation is critical toward maternal effects on gene expression and generate phenotypic differentiation of offspring which resulted in behavioral changes across generations (Champagne & Curley, 2009). Previous cellular and animal studies also reported that changes in neurons are related to an epigenetic alterations that may contribute to the higher variations of DNA methylation within neurons (Iwamoto et al., 2011; McCoy et al., 2019). Moreover, the unique changes in BDNF methylation were also associated with the different pattern of exon IV in the memory formation (Day & Sweatt, 2011). Therefore, our finding suggests that epigenetic modifications during transgenerational process involved the alterations of DNA methylation and consequently influenced the variations of genes expression associated with learning and memory functions. However, further studies are required to determine the role of DNA methylation in memory formation and how epigenetic mechanisms may be relevant for cognitive processes.

In conclusion, the improvement learning and memory functions of F1 and F2 mice in the Y‐maze task were due to the synthetized EBN supplementation from their maternal, and this result correlated with an increase in some brain gene expression and synaptic vesicle densities. In addition, it was shown here that the brain gene expression was involved in sialic acid biosynthesis during period of maternal EBN supplementation and transmitted to their generations. Further studies are required to compare sialic acid content in the F1‐ and F2‐generation animals. This may explain how much of the sialic acid content from the maternal EBN supplementation can truly be considered as transmitted across several generation offspring.

## CONFLICT OF INTEREST

The authors declare no conflict of interest.

## AUTHOR CONTRIBUTION

HA and SAA designed the experiments. OM, MAPR, MJE, and NMH performed the experiments and contributed to the collection, analysis, and interpretation of the data. HA and OM drafted the manuscript, and revised by MHMN, HAH, and SAA. All authors provided the approval for publication of the manuscript.

### Peer Review

The peer review history for this article is available at https://publons.com/publon/10.1002/brb3.1817.

## Data Availability

The data that support the findings of this study are available on request from the corresponding author. The data are not publicly available due to privacy or ethical restrictions.
